# Systematic Comparison of Cell Wall-Related Proteins of Different Yeasts

**DOI:** 10.3390/jof7020128

**Published:** 2021-02-09

**Authors:** Mateja Lozančić, Bojan Žunar, Dora Hrestak, Ksenija Lopandić, Renata Teparić, Vladimir Mrša

**Affiliations:** 1 Laboratory of Biochemistry, Faculty of Food Technology and Biotechnology, University of Zagreb, 10000 Zagreb, Croatia; mlozancic@pbf.hr (M.L.); bzunar@pbf.hr (B.Ž.); dhrestak@pbf.hr (D.H.); rteparic@pbf.hr (R.T.); 2 Department of Biotechnology, University of Natural Resources and Applied Life Sciences, 1180 Vienna, Austria; ksenija.lopandic@boku.ac.at

**Keywords:** cell wall, yeast cell wall, cell wall proteins, cell wall proteome, cell wall evolution

## Abstract

Yeast cell walls have two major roles, to preserve physical integrity of the cell, and to ensure communication with surrounding molecules and cells. While the first function requires evolutionary conserved polysaccharide network synthesis, the second needs to be flexible and provide adaptability to different habitats and lifestyles. In this study, the comparative *in silico* analysis of proteins required for cell wall biosynthesis and functions containing 187 proteins of 92 different yeasts was performed in order to assess which proteins were broadly conserved among yeasts and which were more species specific. Proteins were divided into several groups according to their role and localization. As expected, many *Saccharomyces cerevisiae* proteins involved in protein glycosylation, glycosylphosphatidylinositol (GPI) synthesis and the synthesis of wall polysaccharides had orthologues in most other yeasts. Similarly, a group of GPI anchored proteins involved in cell wall biosynthesis (Gas proteins and Dfg5p/Dcw1p) and other non-GPI anchored cell wall proteins involved in the wall synthesis and remodeling were highly conserved. However, GPI anchored proteins involved in flocculation, aggregation, cell separation, and those of still unknown functions were not highly conserved. The proteins localized in the cell walls of various yeast species were also analyzed by protein biotinylation and blotting. Pronounced differences were found both in the patterns, as well as in the overall amounts of different groups of proteins. The amount of GPI-anchored proteins correlated with the mannan to glucan ratio of the wall. Changes of the wall proteome upon temperature shift to 42 °C were detected.

## 1. Introduction

As single cell organisms, microbial cells are confronted with changes in their habitats that include variations in osmolyte concentrations. To preserve cellular integrity, cells form external envelopes, cell walls, which are able to withstand differences in osmotic pressure between the inner space and the surrounding. Principally, this role is accomplished by building a polysaccharide network with required physical and chemical properties. While in bacteria this moiety is composed of peptidoglycan, yeast cell walls contain more complex structures consisting of β-1,3-glucan with lesser amounts of β-1,6-glucan and chitin [1]. Therefore, the enzymatic apparatus required for the formation of fungal cell walls is more complex than the one in bacterial cells. Besides, yeast cells undergo different morphological changes in the cell’s lifetime such as budding, shmooing, or sporulation. These changes require additional enzymes for the remodeling of the carbohydrate network, but it should be noted that the growth of the cell itself requires flexibility of the cell wall. This flexibility is accomplished by an equilibrium of enzymatic activities of different glycosidases and transglycosidases that shifts towards building, or decomposition of the walls, depending on the temporary and spatial requirements [2,3,4,5,6,7]. All these processes cause the necessity of a much larger set of proteins in the fungal than in bacterial cell walls. Additionally, the cell wall fulfils additional roles in communication of the fungal cell with surrounding cells and matrices as it is the case in for instance mating or flocculation [8,9]. These processes require additional cell wall proteins. Finally, for quite a lot of wall proteins, their physiological roles have not been clarified yet.

Altogether, it has been estimated that between 50 and 60 proteins reside in the cell wall of *Saccharomyces cerevisiae*. A part of these proteins is simply adsorbed non-covalently to β-1,3-glucan [10], while the others are linked covalently. Of the latter, most proteins migrate along the secretory pathway to the plasma membrane in a GPI-anchored form and are then translocated to preformed β-1,6-glucan molecules attached to the β-1,3-glucan network [11,12]. A smaller group of non-GPI bound proteins comprise mostly Pir-proteins covalently attached to β-1,3-glucan through ester bonds created by particular glutamines located in a specific repeating motif at the N-terminal part of the protein [13]. Some proteins seem to be bound to the cell wall by two different mechanisms, like the Scw4 that is partly bound to the wall non-covalently and partly covalently by a so far unexplained reaction [14], or Cwp1 that contains both GPI-anchoring signal and the characteristic Pir sequence [15]. The way of attachment of proteins to β-1,3-glucan defines the possibility of their isolation from the wall. Non-covalently attached proteins are usually extracted by hot SDS, sometimes with the addition of β-mercaptoethanol, GPI-bound proteins by the treatment of purified walls with glucanases, while the Pir-proteins together with the covalently attached Scw4 are extracted by mild alkali. Specific labeling of wall proteins by biotinylation and subsequent extraction of the three groups of proteins enabled their identification in the cell walls of *S. cerevisiae* [16]. In other yeasts, such a systematic analysis of the cell wall proteome has been performed only in *Candida albicans* [17,18], although individual proteins in different yeast genera/species have been isolated and described.

Previous proteomic analyses attempted to identify the subset of cell wall proteins with limited success. The early work of Washburn et al. [19] investigated *Saccharomyces cerevisiae* proteome by so-called multidimensional protein identification technology (MudPIT) including multidimensional liquid chromatography, tandem mass spectrometry, and database searching by the SEQUEST algorithm, identifying 1484 proteins, including some low-abundance proteins and several integral membrane proteins. Lange et al. [20] described the targeted quantitative approach by which predetermined protein sets were first identified and subsequently quantified at high sensitivity. This approach was used by Picotti et al. [21] in an analysis of the *S. cerevisiae* proteome for detecting proteins expressed in concentrations below 50 copies per cell. However, neither of these approaches could be used for protein localization on a large scale. Kumar et al. [22] used directed topoisomerase I-mediated cloning strategies and genome-wide transposon mutagenesis for epitope-tagging 60% of the *Saccharomyces cerevisiae* proteome. They determined the localization of 2744 yeast proteins by high-throughput immunolocalization of tagged proteins. Huh et al. [23] created a collection of yeast strains expressing full-length proteins, tagged at the carboxy terminal end with green fluorescent protein (GFP), and analyzed them by fluorescence microscopy to detect protein subcellular localization. The authors defined localization for 4156 proteins representing 75% of the yeast proteome. However, the cell wall proteins were frequently mislocalized in this study, since C-terminal addition of the GFP affected their C-terminal targeting signals. Insenser et al. [24] combined two proteomic approaches to detect protein subcellular localization. In the first, intact *Saccharomyces cerevisiae* cells were treated with dithiothreitol (DTT), extracted proteins were separated by 2D-PAGE and identified by mass spectrometry. In the second approach, surface proteins of intact cells were digested with trypsin and treated with DTT, and afterwards analyzed by LC–MS/MS. The authors identified 67 proteins with DTT treatment and 52 with DTT and trypsin digestion. However, several detected proteins were atypical for cell walls, and only 20% were common to both protocols. The second-generation approach in which surface-exposed proteins of intact cells were “shaved” by proteases and analyzed by LC/MS/MS was reviewed by Olaya-Abril et al. [25]. However, these methods frequently identified several intracellular proteins within yeast “surfomes” presumably because of the partial permeabilization of plasma membranes. Information resources for *S. cerevisiae* protein localization might be found in Yeast Protein Localization Database (http://ypl.tugraz.at/pages/home.html, (accessed on 26 January 2021)) and Yeast GFP Fusion Localization Database (http://yeastgfp.ucsf.edu, (accessed on 26 January 2021)).

Apart of *S. cerevisiae* cell wall proteome, the wall proteomes of opportunistic human pathogens *Candida albicans* [26], *Candida parapsilosis* and *Candida tropicalis* [27], as well as *Kluyveromyces lactis* [28] and *Schizosaccharomyces pombe* [29] were investigated in similar manner by either using combination of specific isolations of protein fractions from isolated cell walls and MS analysis [28,29,30] or cell surface shaving and shotgun proteomic approach [27,31]. Recently some extensive reviews on *C. albicans* cell wall proteomes were published by Gil-Bona et al. [32] and Reyna-Beltran et al. [33].

In this paper, cell wall related proteins of 92 different yeasts were systematically compared to the wall related proteins of *S. cerevisiae* taken as the reference. The analysis included the comparison of all proteins reported to be located in the *S. cerevisiae* cell wall, as well as those not directly residing in the wall but required for the cell wall synthesis, with their orthologues in other fungal genera/species whose genomes have been sequenced and published. In this way, we tried to assess which cell wall proteins were conserved among different yeasts, and which were rather specific for *S. cerevisiae*. The *in silico* analysis has further been supported by the labeling, isolation, and streptavidin/biotin blotting of cell wall proteins of different yeasts and the observed differences in protein patterns have been discussed.

## 2. Materials and Methods

### 2.1. Comparison of Cell Wall Proteomes Using Gene Prediction and Orthologue Annotation

**Figure 1:**
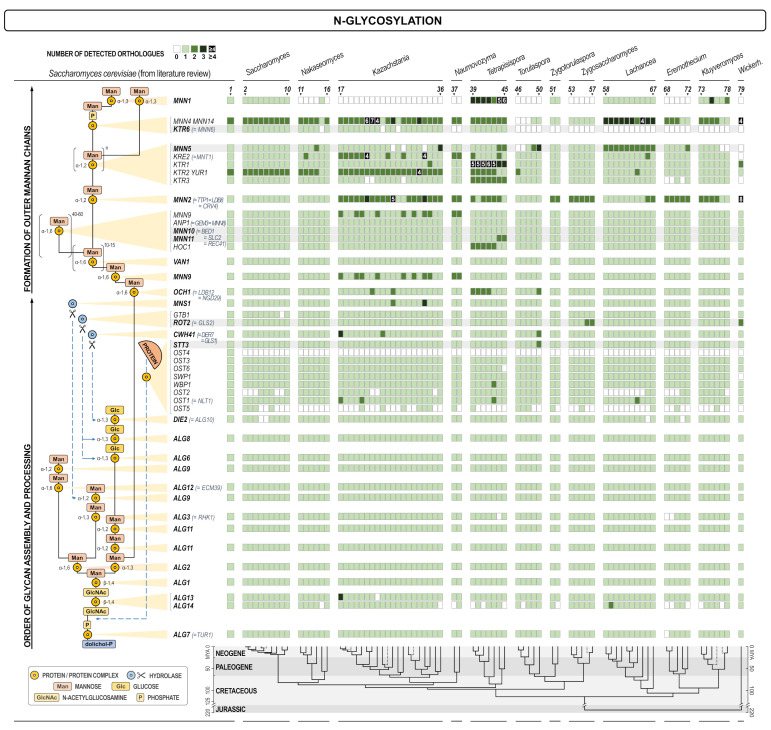

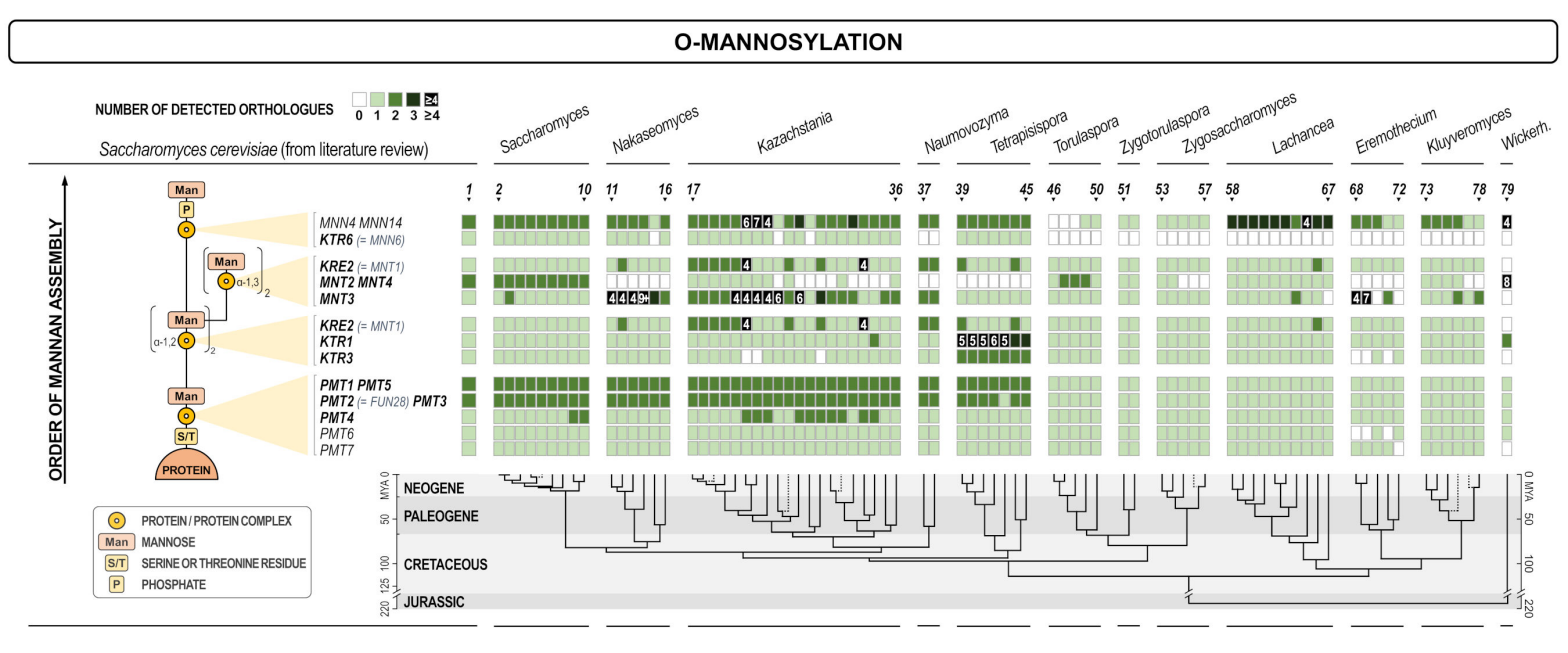
Heat map of orthologues of *S. cerevisiae* genes involved in protein glycosylation. Every gene is presented in one row, while every yeast is presented in one line. Compared yeasts are: 1. *Saccharomyces (S.) cerevisiae* in SGD; 2. *S. cerevisiae* re-annotated; *S. cerevisiae var. boulardii*; 4. *S. paradoxus*; 5. *S. mikatae*; 6. *S. jurei*; 7. *S. kudriavzevii*; 8. *S. arboricola*; 9. *S. uvarum*; 10. *S. eubayanus*; 11. *Candida (C.) nivariensis*; 12. *Nakaseomyces (Na.) delphensis*; 13. *C. bracarensis*; 14. *C. glabrata*; 15. *Na. bacillisporus*; 16. *C. castellii*; 17. *Kazachstania (Ka.) solicola*; 18. *Ka. aerobia*; 19. *Ka. servazzii*; 20. *Ka. unispora*; 21. *Ka. siamensis*; 22. *Ka. transvaalensis*; 23. *Ka. yakushimaensis*; 24. *Ka. taianensis*; 25. *Ka. naganishii*; 26. *Ka. telluris*; 27. *Ka. bromeliacearum*; 28. *Ka. intestinalis*; 29. *Ka. martiniae*; 30. *Ka. turicensis*; 31. *Ka. saulgeensis*; 32. *Ka. kunashirensis*; 33. *Ka. spencerorum*; 34. *Ka. rosinii*; 35. *Ka. africana*; 36. *Ka. viticola*; 37. *Naumovozyma (Nau.) dairenensis*; 38. *Nau. castellii*; 39. *Tetrapisispora (Te.) namnaonensis*; 40. *Te. fleetii*; 41. *Te. phaffii*; 42. *Te. iriomotensis*; 43. *Vanderwaltozyma polyspora*; 44. *Te. blattae*; 45. *Yueomyces sinensis*; 46. *Torulaspora (To.) franciscae*; 47. *To. pretoriensis*; 48. *To. delbrueckii*; 49. *To. maleeae*; 50. *To. microellipsoides*; 51. *Zygotorulaspora (Zyt.) florentina*; 52. *Zyt. mrakii*; 53. *Zygosaccharomyces (Zys.) bisporus*; 54. *Zys. bailii*; 55. *Zys. kombuchaensis*; 56. *Zys. mellis*; 57. *Zys. rouxii*; 58. *Lachancea (L.) lanzarotensis*; 59. *L. meyersii*; 60. *L. dasiensis*; 61. *L. nothofagi*; 62. *L. quebecensis*; 63. *L. thermotolerans* 64. *L. waltii*; 65. *L. mirantina*; 66. *L. fermentati*; 67. *L. kluyveri*; 68. *Eremothecium (E.) gossypii*; 69. *Ashbya aceri*; 70. *E. cymbalariae*; 71. *E. coryli*; 72. *E. sinecaudum*; 73. *Kluyveromyces (Kl.) lactis*; 74. *Kl. dobzhanskii*; 75. *Kl. marxianus*; 76. *Kl. wickerhamii*; 77. *Kl. nonfermentans*; 78. *Kl. aestuarii*; 79. *Wickerhamomyces anomalus*. The scheme of the cellular process with the site in which certain gene is involved is shown on the left. The intensity of the color of every square is proportional to the number of orthologues found as depicted in the inset legend. White square means no orthologue has been found. The lower part of the figure presents the evolutionary chronogram of each species. Genes coding for catalytic subunits are presented in bold and have a grey background if they are parts of a bigger complex.

**Figure 2:**
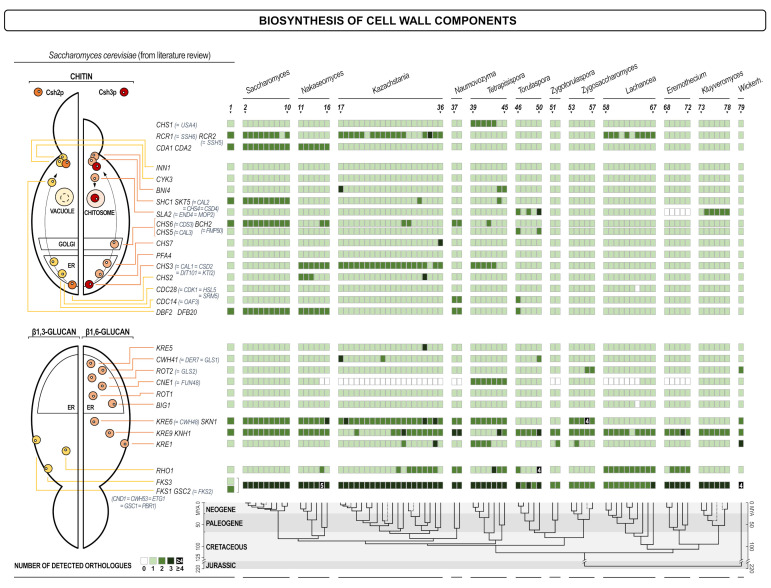
Heat map of orthologues of *S. cerevisiae* genes involved in the synthesis of cell wall polysaccharides. Every gene is presented in one row, while every yeast (see the legend of Figure 1) is presented in one line. The scheme of the cellular process with the site in which the product of each gene is involved is shown on the left. The intensity of the color of every square is proportional to the number of orthologues found as depicted in the inset legend. White square means no orthologue has been found. The lower part of the figure presents the evolutionary chronogram of each species.

**Figure 3:**
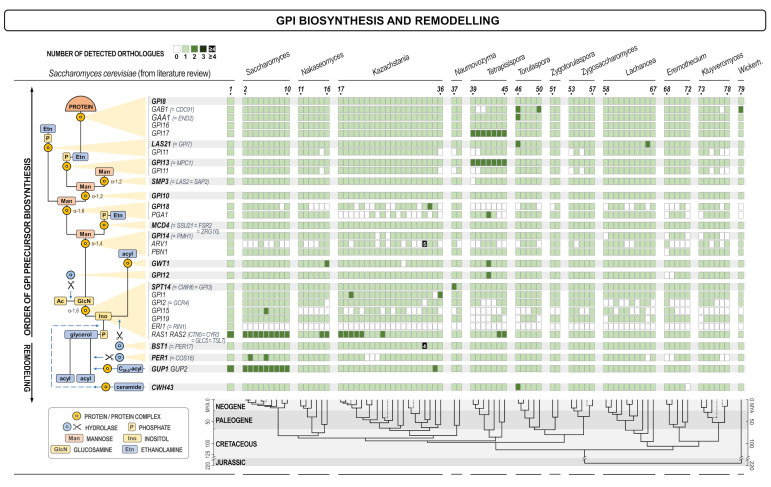
Heat map of orthologues of *S. cerevisiae* genes involved in the synthesis of GPI and the GPI-protein conjugates. Every gene is presented in one row, while every yeast (see the legend of Figure 1) is presented in one line. The scheme of the cellular process with the site in which the product of each gene is involved is shown on the left. The intensity of the color of every square is proportional to the number of orthologues found as depicted in the inset legend. White square means no orthologue has been found. The lower part of the figure presents the evolutionary chronogram of each species. Genes coding for catalytic subunits are presented in bold and have a grey background if they are parts of a bigger complex.

**Figure 4:**
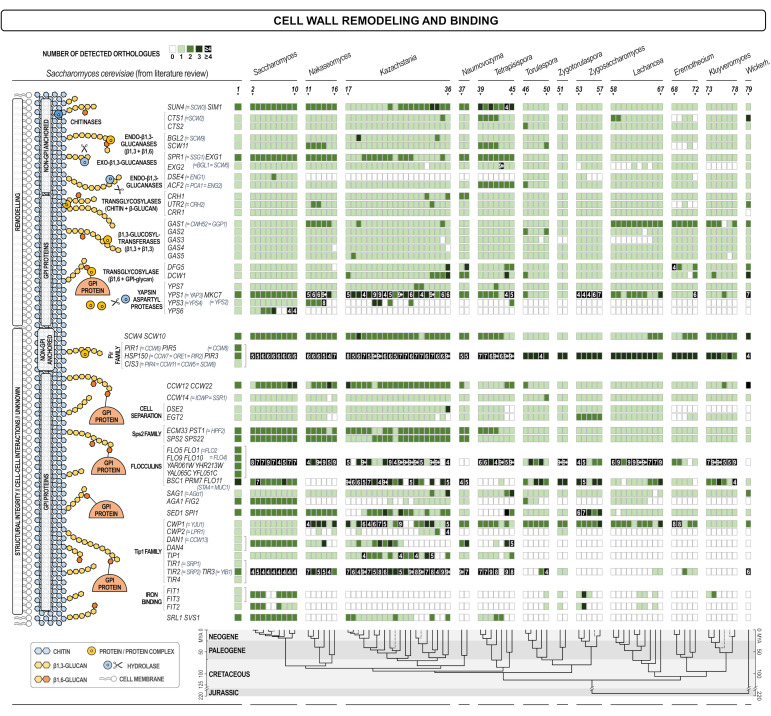
Heat map of orthologues of *S. cerevisiae* genes coding for proteins that reside in the cell wall. Every gene is presented in one row, while every yeast (see the legend of Figure 1) is presented in one line. The scheme of the cellular process with the site in which the product of each gene is involved is shown on the left. The intensity of the color of every square is proportional to the number of orthologues found as depicted in the inset legend. White square means no orthologue has been found. The lower part of the figure presents the evolutionary chronogram of each species.

To ensure uniform genome annotation, the genome of each species was re-annotated using AUGUSTUS v3.1 [34], with the gene model based on *Saccharomyces cerevisiae* S288C and the parameters that allowed for the existence of overlapping genes on the opposite DNA strands; other settings defaulted to their preset values. Analyzed species were chosen due to their phylogenetic proximity to *S. cerevisiae*, inferred from Shen et al. [35], the work which also served as the source of the chronogram at the bottom of Figure 1, Figure 2, Figure 3 and Figure 4.

Orthologous clusters were defined with OrthoVenn2 [36], using an inflation value of 1.5 to determine the cluster structure. As suggested previously [37], an E-value cut-off of 10^−5^ was used to calculate pairwise genome comparisons and annotate uncovered clusters. For this purpose, analyzed species were grouped into batches of up to seven species. OrthoVenn2 identified clusters in each batch, comparing them with the official *S. cerevisiae* gene annotation (Saccharomyces Genome Database R64-2-1), with the gene being deemed an orthologue of an *S. cerevisiae* gene if it clustered with it or if the OrthoVenn2’s Swiss-Prot hit identified it as such. When the orthologue number varied across the clade, the discrepancy was inspected with ‘BLASTP’ implemented in OrthoVenn2 and manually curated, accounting for the E-value, per cent identity, and per cent query cover of the hits. The list of yeast strains used in the in silico analysis of the cell wall proteomes is given in Table S1.

### 2.2. Labeling of Cell Wall Proteins

Yeasts listed in Table S2. were grown to the early exponential growth phase in yeast malt extract medium (3 g/L yeast extract, 3 g/L malt extract, 5 g/L peptone) containing 2% glucose (*w*/*v*) as a carbon source, at 30 °C (except for *Metschnikowia* species which were grown at 24 °C). Cells were harvested and washed twice with deionized water and twice with 50 mM K-phosphate buffer pH 8.0. Cells were then resuspended in the same buffer containing 0.5 mg/mL of EZ-Link Sulfo-NHS-LC-LC-Biotin reagent (Thermo Scientific, Waltham, MA, USA) and incubated on ice for 90 min. After that, cells were washed twice with 50 mM Tris-MgCl_2_ buffer and twice with 50 mM K-phosphate buffer pH 8.0.

All yeasts were obtained from the ACBR microbial culture collection at the Department of Biotechnology, University of Natural Resources and Life Sciences, Vienna.

### 2.3. Isolation of Cell Wall Proteins

Cells were resuspended in the 50 mM K-phosphate buffer pH 8.0, broken mechanically with glass beads using BeadBug homogenizer (Benchmark Scientific, Inc., Edison, NJ, USA), and washed four times with the 50 mM K-phosphate buffer pH 8.0. Proteins were extracted from isolated cell walls in three different manners. Non-covalently bound proteins were extracted by boiling cell walls for 10 min in the Laemmli buffer (0.0625 M Tris-HCl buffer pH 6.8, 2% SDS; 5% *v*/*v* β-mercaptoethanol; 0.001% bromphenol blue). To remove any remaining non-covalently bound proteins, this step was repeated two more times. After that, cell walls were washed four times in the 50 mM K-phosphate buffer pH 8.0 and then twice in deionized water. Remaining cell wall proteins were extracted by the incubation in 30 mM NaOH overnight at 4 °C, or by the incubation in 50 mM K-phosphate buffer pH 6 with the addition of 9U of β-1,3-glucanase 81A (NZYTech, Lisbon, Portugal) for 2h at 55 °C. Extracted proteins were subjected to electrophoresis and detected on blots using streptavidin-horseradish peroxidase conjugate.

To prepare samples that would be directly comparable, the amount of each extract used for electrophoresis was normalized to the wet mass of cell walls measured prior to extraction of non-covalently bound proteins. The wet mass of cell walls was within the range from 80 mg to 150 mg. The volume of the extraction buffer for each type of extraction corresponded to the mass of isolated cell walls. Final concentration of cell walls for the extraction with NaOH, or β-1,3-glucanase 81A was 1 mg/µL, while for the extraction with Laemmli buffer it was 0.1 mg/µL. Each experiment was repeated twice with samples originating from two independent growths.

### 2.4. Electrophoresis and Blotting

Electrophoresis was performed by the method of Laemmli [38] using 4% stacking and 12% polyacrylamide resolving gels. To visualize biotinylated proteins, the proteins were blotted to PDVF membrane which was then incubated for 1 h in 10 mL of blocking buffer (50 mM Tris–HCl pH 7.5, 0.15 M NaCl, 0.1% *v*/*v* Triton X-100) with 5% *w*/*v* BSA, then 1 h in the same buffer with the addition of Pierce streptavidin-horseradish peroxidase conjugate (Thermo Scientific, Waltham, MA, USA). Finally, blots were washed three times with the blocking buffer and developed using the ECL kit (BioRad, Hercules, CA, USA). Visualization was performed using C-digit blot scanner (LI-COR Biosciences, Lincoln, NE, USA) and obtained images were analyzed using Image Studio software (LI-COR Biosciences, Lincoln, NE, USA).

### 2.5. Carbohydrate Analysis

Composition of polysaccharides in cell walls of different yeasts was determined according to Schweigkofler et al. [39].

## 3. Results

### 3.1. In Silico Analysis of Cell Wall Related Proteins of Different Yeasts

Previous *in silico* comparison of genes coding for 187 “cell wall related proteins” of *S. cerevisiae* with 17 other fungi showed that the number of orthologues found correlated with the evolutionary distance of individual fungi from the *S. cerevisiae* taken as the standard [40]. In this work we have performed a much more extensive comparison of 187 proteins related to *S. cerevisiae* cell wall with proteomes of 92 different yeasts, 77 of which were rather closely related to *S. cerevisiae*, while 15 were genetically more distant to the comparator. To uncover differences in the cell wall biogenesis and related cellular processes like protein glycosylation in yeasts close to *S. cerevisiae*, we first searched for orthologues of relevant genes in publicly available genomes of 77 species that diverged from *S. cerevisiae* up to 120 million years ago (Table S1).

To prevent the differences in existing annotations from introducing spurious variation in the analysis, we uniformly re-annotated each of the genomes using OrthoVenn2. When applied on the genome of *S. cerevisiae* S288C, our re-annotation identified 187 out of 191 genes contained in the *Saccharomyces genome database* (SGD) involved in protein glycosylation and cell wall remodeling, thus confirming the effectiveness of the approach. Four genes that have not been found were *ARV1*, and *ERI1* that have putative functions in GPI biosynthesis, and *OST2*, and *OST4*, parts of the oligosaccharyl transferase complex. All these genes are rather small and therefore escaped re-annotation. Altogether, 13,402 genes were recognized as involved in protein glycosylation and cell wall biogenesis in all 77 yeasts (Supplementary Materials, Table S1), making up to 2.8% of the total of 471,884 putative genes in all species. We also compared the *S. cerevisiae* cell wall related genes to those of the 13 yeasts that were taxonomically more distant although these results have to be taken with caution since in some cases proteomes of these yeasts were most probably incomplete.

As expected, proteins involved in protein glycosylation have been found to be rather conserved throughout the whole spectrum of yeasts (Figure 1). The exceptions were *OST2*, *OST4*, and *OST5* subunits of oligosaccharyl transferase (*OST2* and *OST4* failed to be re-annotated), a large complex that transfers core oligosaccharides from dolichol phosphate to asparagine in the N-glycosylation pathway, and two genes involved in the synthesis of the outer mannan chains in Golgi (*MNN1* and *MNN6/KTR6*). Both functions are essential in *S. cerevisiae*, thus they have to be performed in other yeasts by proteins that are less similar and were not identified as orthologues. As presented in Figure 1 (lower panel), O-mannosylation machinery shows even less variability than the N-glycosylation pathway. Similarly, proteins involved in the biosynthesis of β-1,3-, and β-1,6-glucan, as well as chitin (Figure 2) were also very conserved as expected considering that all three polysaccharides were essential in the wall and that their structure was of highest importance for the cell integrity. An exception within this group is the calnexin gene *CNE1*. Interestingly, calnexin is a protein conserved among higher eukaryotes, yet several yeast genera do not have this protein. In several cases proteins with high structural homology were presented together in Figure 2, for instance *SHC1* and *SKT5*. When considered separately, however, orthologues of the protein Shc1 were rather seldom found, while Skt5 was broadly represented, indicating that *SHC1* probably diverged more in *S. cerevisiae*. Functionally, these two proteins were structural and functional homologs (chitin synthase III activator in the sporulation process) [41].

Higher variability was observed among proteins identified in the glycosylphosphatidylinositol biosynthesis and remodeling pathway (Figure 3). Apart of the two small genes *ARV1* and *ERI1* that were not identified in the re-annotation of the *S. cerevisiae* S288c from the SGD, we found no orthologues of *GPI18*, *PGA1*, and *GPI15* in quite a few species/genera. Besides, *GPI2* and *GPI19* had no orthologues in yeasts that were evolutionarily more distant to *S. cerevisiae*, and several other genes were not found in individual yeast species (Figure 3). Interestingly, proteins Pga1 and Gpi18 are members of the α-1,6-mannosyl transferase complex required for the addition of the second mannose in the formation of the GPI anchor, and are both essential in *S. cerevisiae*. The proteins Gpi15, Gpi2, and Gpi19 are involved in the biosynthesis of the core GPI molecule and they are also essential in *S. cerevisiae*. Thus, our results indicate that these two particular steps in the biosynthesis of GPI are performed by proteins with less homology to the complexes found in *S. cerevisiae*. In other steps, however, in most yeasts *S. cerevisiae* orthologues were identified.

Figure 4 presents the analysis of genes whose products are located in the cell wall, or are anchored to the outer side of the plasma membrane expressing their activities in the wall. The group is named “cell wall integrity and remodeling” but it has to be noted that the real physiological activity of quite many of these proteins is still unknown. They could be further divided into several sub-groups according to the way they were incorporated in the wall and their physiological function (if known) that was related to the way of their incorporation in the wall.

The first sub-group contained non-covalently attached proteins. There was a high degree of conservation of these proteins among analyzed yeasts, and with the exception of the *ENG1/DSE4*, all other genes had orthologues in most yeasts. Eng1 is the endoglucanase required for detachment of the daughter cell, therefore its activity must be substituted by other glucanases in other budding yeasts. It could be seen that the number of orthologues decreases with the evolutionary distance from the *Saccharomyces* genus, especially in genera *Lachancea, Eremothecium*, and *Wickerhamii*.

The second sub-group comprised GPI-anchored proteins of the Crh and Gas families of transglycosidases involved in the remodeling of chitin and β-1,3-glucan, respectively. These proteins have important roles in the biosynthesis of the polysaccharide moiety of the wall and probably transfer parts of chitin (Crh), or β-1,3-glucan (Gas) synthesized by chitin and glucan synthases to the existing wall network, thus liberating synthases for further extension of the growing chains. As expected, due to their essential roles, these two families were present in all yeasts although not all members were present in all species (Figure 4).

The third sub-group contained yapsins, a group of aspartic proteases reported to be located at the cell surface. *S. cerevisiae* has five yapsin genes, *YPS1, YPS2, YPS3/YPS4, YPS6*, and *YPS7* [42,43,44,45]. The Yps1 protein was found to be a GPI anchored protein located in the cell wall, while the exact localization of other yapsins is not completely clear but they were reported to be responsible for proteolytic processing and activation of several cell wall proteins [14,46]. The method used for orthologue finding in this work did not recognize orthologues of yapsins *YPS3* and *YPS6* in many species, but it has identified several orthologues of *YPS1*. Thus, all yeasts contained yapsins, but in most cases their protein sequences were more similar to Yps1 than to Yps3 and Yps6.

The fourth sub-group comprised non-GPI covalently attached proteins including those of the Pir protein family that shared characteristic repetitive sequence required for their covalent attachment to β-1,3-glucan. Additionally, two homologous proteins Scw4 and Scw10 that were first identified as non-covalently attached to the wall were subsequently found to contain a fraction that was covalently linked to glucan. These two proteins contain motifs that resemble Pir repetitive sequences but the exact way of their incorporation into the wall is still under investigation. Physiological role of Pir-proteins is not known, while Scw4 and Scw10 share sequence similarity to glucanases, but their enzymatic activity has not been established. Orthologue analysis of this group revealed that it was present in all analyzed yeasts and that most of them contained several genes of the Pir family. Since these genes share a high degree of homology/identity it was not possible to differentiate orthologues of individual PIR genes, but they were taken as a group.

Finally, the fifth sub-group contained a series of GPI-anchored proteins of various roles among which some had functions in cell-cell communication (flocculation, agglutination), some in iron binding (Fit proteins), but most have so far unknown physiological roles. In Figure 4 it can be seen that the variability within this group of proteins is the highest. We were not able to identify orthologues of quite several *S. cerevisiae* genes and the variability increased with the evolutionary distance between the species/genera. Thus, although the attachment of these proteins requires a rather complicated, energy consuming, and highly conserved GPI synthesis, proteins attached are much less conserved and are most probably those that reflect the life-style or habitat adaptation characteristics.

**Figure 5:**
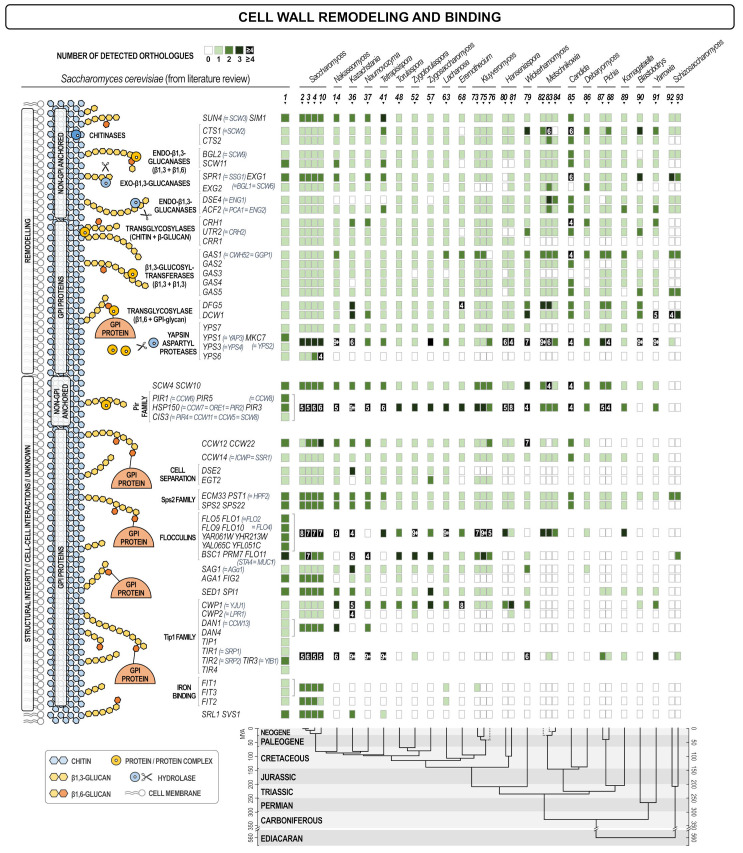
Heat map of orthologues of *S. cerevisiae* genes coding for proteins that reside in the cell wall of yeasts that are taxonomically more distant from *S. cerevisiae*. Every gene is presented in one row, while every yeast is presented in one line. The heat map includes representatives of genera presented in Figure 4 for comparison with evolutionary more distant yeasts: 80 *Hanseniaspora (H.) uvarum,* 81 *H. osmophila*, 82 *Metschnikowia (M.) reukaufii*, 83 *M. pulcherrima*, 84 *M. bicuspidata*, 85 *Candida albicans*, 86 *Debaryomyces hansenii*, 87 *Pichia (P.) kudriavzevii*, 88 *P. membranifaciens*, 89 *Komagataella pastoris*, 90 *Blastobotrys adeninivorans*, 91 *Yarrowia lipolytica*, 92 *Schizosaccharomyces (Schiz.) japonicus*, 93 *Schiz.* *pombe*. The scheme of the cellular process with the site in which the product of each gene is involved is shown on the left. The intensity of the color of every square is proportional to the number of orthologues found as depicted in the inset legend. White square means no orthologue has been found. The lower part of the figure presents the evolutionary chronogram of each species.

When the orthologue search of the cell wall localized proteins has been extended to yeasts that were taxonomically further away from *S. cerevisiae* the negative correlation between the evolutionary distance and the number of orthologues was even more pronounced (Figure 5). Although the accessible genomes were not always complete and sometimes contained presumable errors, it was apparent that there was a pronounced difference between the non-covalently bound proteins that were significantly more conserved, and the more species-specific GPI-anchored proteins. Interestingly, most non-covalently bound proteins had orthologues in budding yeasts but the two *Schizosaccharomyces* species contained only orthologues of Bgl2, Exg1, and Sun4. *Schiz. pombe*, but not *Schiz. japonicus* also contained a protein homologue to a putative endoglucosidase Dse4. Both paralogs, Exg1 and Spr1 are probably exoglucanases, but it is not clear whether they can also serve as functional homologues. Interestingly, orthologues of Spr1 could only be found in several yeasts, mostly taxonomically closer to *S. cerevisiae*. Besides Spr1, the only non-covalently bound protein that was rather specific for *S. cerevisiae* and closely related species was Srl1, a protein of unknown function.

The comparison of GPI-anchored proteins revealed again that they were generally species specific. Exceptions were Ecm33, Pst1, and members of the Gas family. These proteins, although conserved, have very different functions. Ecm33 and Pst1 seem to be paralogs but their function is not known [47,48]. As already mentioned, the proteins of the Gas family are glucosyl transferases that seem to be involved in the formation of the β-1,3-glucan moiety [49,50]. Additionally, most yeasts (but not all) had an orthologue of either Crh1, or Crh2 (Utr2). These two proteins are chitin transferases that transfer chitin chains to the non-reducing ends of either β-1,3-, or β-1,6-glucan [51,52]. All other *S. cerevisiae* GPI-anchored proteins had their equivalents only in few other yeasts. It should be noted that in some yeasts flocculation Flo proteins had more (up to 18) homologous equivalents. Yapsins were found in all yeasts except *Schiz. pombe*, usually as a gene family showing that their role was essential for the wall formation, or remodeling.

Pir proteins were present in all yeasts except in *Debaryomyces hansenii*, *Blastobotrys adeninivorans*, as well as in fission yeasts. Since the role of these proteins is unknown, it is difficult to speculate why they do not need this protein family.

### 3.2. Streptavidin/Biotin Blot Analysis of Cell Wall Proteomes of Different Yeasts

**Figure 6:**
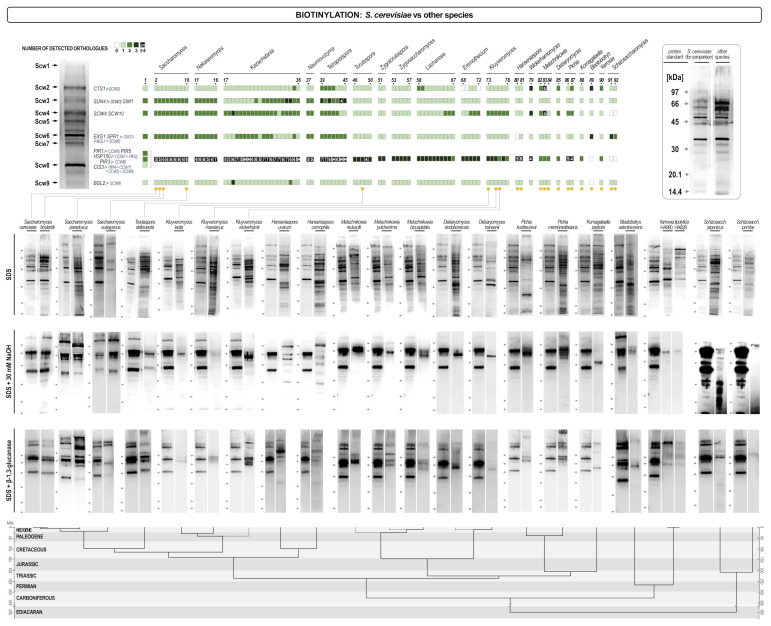
Streptavidin/biotin blots of cell wall proteins of different yeasts extracted by hot SDS (non-covalently attached proteins), alkalis (Pir proteins + Scw4), and glucanases (GPI-anchored proteins). The upper part of the figure helps placing analyzed yeasts in the orthologues heat map in Figure 5. Every blot is presented in pair with the corresponding blot of *S. cerevisiae* (left) for comparison. The lower part of the figure presents the evolutionary chronogram of each species. Position of protein molecular mass markers is indicated on the left of each blot.

*In silico* analysis of cell wall related proteins showed differences in the genomic potential of individual species to synthesize cell wall proteins. However, it does not provide information on the actual situation in the cell wall of different yeasts. Labeling of cell wall proteins by biotinylation and the subsequent streptavidin/biotin blot analysis of labeled proteins provided specific protein patterns that served for identification of several *S. cerevisiae* cell wall proteins [16]. Proteins are incorporated in the cell wall in at least three different ways. Non-covalently attached proteins could be extracted by hot SDS, Pir proteins together with a part of Scw4 using mild alkalis, while GPI-anchored proteins could be released from the cell wall by the digestion with β-1,3- glucanases. Therefore, the three extracts combined contained most if not all cell wall proteins expressed under specific growth conditions [16]. Streptavidin/biotin blot analysis (Figure 6) showed that there was a pronounced difference both in the protein patterns of different yeast species, and in the quantity of proteins present in their cell walls. Like in the *in silico* analysis, yeasts that were taxonomically closer to *S. cerevisiae* had more similar protein patterns. *S. cerevisiae var. bulardii* did not differ in its cell wall protein composition from *S. cerevisiae*, neither in the pattern of non-covalently attached proteins, nor in the composition of alkali extractable, or GPI-linked proteins. *S. paradoxus* and *S. eubayanus* already showed distinct differences in protein patterns, to some extent also present in *Torulaspora delbrueckii*. The three *Kluyveromyces species* tested (*K. marxianus, K. lactis*, and *K. wickerhamii*) already differed significantly in all three extracts. The amounts of non-covalently attached cell wall proteins did not differ too much from the *S. cerevisiae* control, but the pattern was significantly different. In the NaOH extract (Pir proteins + Scw4) only one clear distinct band could be detected at the approximate size of the Scw4, indicating that although these yeasts have two genes for Pir proteins, their presence in the wall could probably not be detected. Also, very few GPI-anchored proteins could be seen in the glucanase extract and the amount of proteins was very different among species. To some extent similar results were obtained when three *Pichia/Komagataella* species were analyzed (*P. kudriavzevii, P. membranifaciens* and *K. pastoris*). Again, SDS extracts differed significantly in their composition but not too much in the overall amount of proteins, while in the NaOH extract only one strong band was detected. In *P. kudriavzevii* and in *P. membranifaciens* it was of the approximate size of Scw4, while in *K. pastoris* the protein was smaller and it was not possible to identify it without sequencing. *P. kudriavzevii* has two *PIR* genes, and *P. membranifaciens* one. Glucanase extracts were qualitatively more similar, although still bearing significant differences to *S. cerevisiae*, but *P. pastoris*, and *P. kudriavzevii* had significantly less, while *P. membranifaciens* had even somewhat more GPI-anchored proteins than *S. cerevisiae*. In two *Hanseniaspora* species analyzed (*H. uvarum* and *H. osmophila*) the patterns of non-covalently attached proteins were comparable, although not identical to *S. cerevisiae*. Interestingly, in the NaOH extract more bands of strong intensity have been detected although *H. uvarum* had two, and *H. osmophila* only one *PIR* gene. Besides, *H. uvarum* is one of the very few yeasts that does not have the *SCW4* gene, nor its functional homolog *SCW10*. Finally, both species had amounts of GPI anchored proteins comparable to *S. cerevisiae*, but their pattern was different as expected having in mind differences in this group of proteins recorded in the *in silico* analysis. Two *Yarrowia lipolytica* strains have been analyzed and the *Y. lipolytica* HA990 had a similar pattern of non-covalently attached proteins corroborating similarities found in the genome analysis (Figure 5). Interestingly, the strain HA826 had a different ratio of band intensities (although the band pattern was not too different from that of HA990) indicating different regulation of wall protein genes. Both strains had many fewer proteins, both in the NaOH extract (again with only one visible band at the size of Scw4), and in the glucanase extract. Three species of *Metschnikowia* (*M. bicuspidata, M. reukaufii*, and *M. pulcherrima*) were analyzed. *In silico* analysis of the *M. bicuspidata* proteome indicated some, but not too many differences in non-covalently attached proteins. Indeed, the streptavidin/biotin blot analysis showed that the protein pattern was comparable, although not identical to the pattern of *S. cerevisiae*. The three *Metschnikowia* species had similar, although not completely identical protein compositions of proteins in the SDS extract. The pattern was clearly different from the one of *S. cerevisiae*. In the alkali extracts one strong band dominated at the size of the Scw4 paralog, while the Pir proteins were hardly (if at all) visible. Glucanase extracts were different in pattern (again following differences found in the genome analysis) but not too much in intensity. One strain of *Torulaspora delbrueckii* has also been analyzed. Although according to the *in silico* analysis it contained orthologues of almost all non-covalently attached proteins the streptavidin/biotin blot pattern was quite different from the one of *S. cerevisiae*. In the alkali extract both Scw4 and the only Pir protein were visible although at a much lower intensity than in *S. cerevisiae*, while the pattern of GPI-anchored proteins was quite similar to that of *S. cerevisiae* despite the large difference of the number of orthologues (Figure 5). *Blastobotrys adeninivorans* was one of the yeasts which showed pronounced differences in the in silico analysis. Indeed, the pattern of non-covalently attached proteins was different, although the amount of proteins was comparable to that of *S. cerevisiae*. In contrast, *B. adeninivorans* had the lowest concentration of both alkali-, and glucanase-extractable proteins of all analyzed yeasts. Two species of *Debaryomyces* (*D. hansenii* and *D. vindobonensis*) were analyzed, as well. *D. hansenii* had orthologues of most non-covalently attached proteins but very few of the GPI-anchored proteins. However, the pattern of SDS-extractable proteins was quite distinct from the one of *S. cerevisiae*, while in the NaOH extract only one strong band was visible at the size of Scw4 (*D. hansenii*, and presumably *D. vindobonensis* do not have Pir proteins). The two species differed very much in the quantity of GPI-anchored proteins. Finally, we also analyzed cell wall proteins of two fission yeasts, *Schizosaccharomyces pombe* and *Schizosaccharomyces japonicus*. As expected, patterns of non-covalently attached proteins were very different from the one of *S. cerevisiae* (although quite similar between the species). Interestingly, although neither of the species have neither Pir proteins, nor Scw4, proteins were detected in the alkali extracts of the wall, particularly strong in case of *Schiz. japonicus*. The pattern of GPI- anchored proteins differed both qualitatively and quantitatively, as expected.

GPI-anchored proteins were reported to be the primary target for N-glycosylation forming the outer mannan layer of the cell. Thus, it can be assumed that yeasts that had less GPI-anchored proteins on streptavidin/biotin blots, also had less mannan added to their surfaces. To test this, we determined the glucan/mannan ratio in the cell walls of three yeasts with least GPI-anchored proteins. *S. cerevisiae* taken as the control had the glucose to mannose ratio of about 1.4. *Blastobotrys adeninivorans* had 61% glucose, 30% mannose, and 9% galactose, thus the ratio of glucose to mannose+galactose was around 1.6 (or glucose to mannose alone 2.0). *Debaryomyces hansenii* had a glucose to mannose ratio 1.95, while *D. vindobonensis* had the ratio around 1.8. Therefore, we could confirm the correlation between the amount of GPI-anchored proteins in the wall with the amount of mannan and presumably the thickness of the mannan layer.

**Figure 7:**
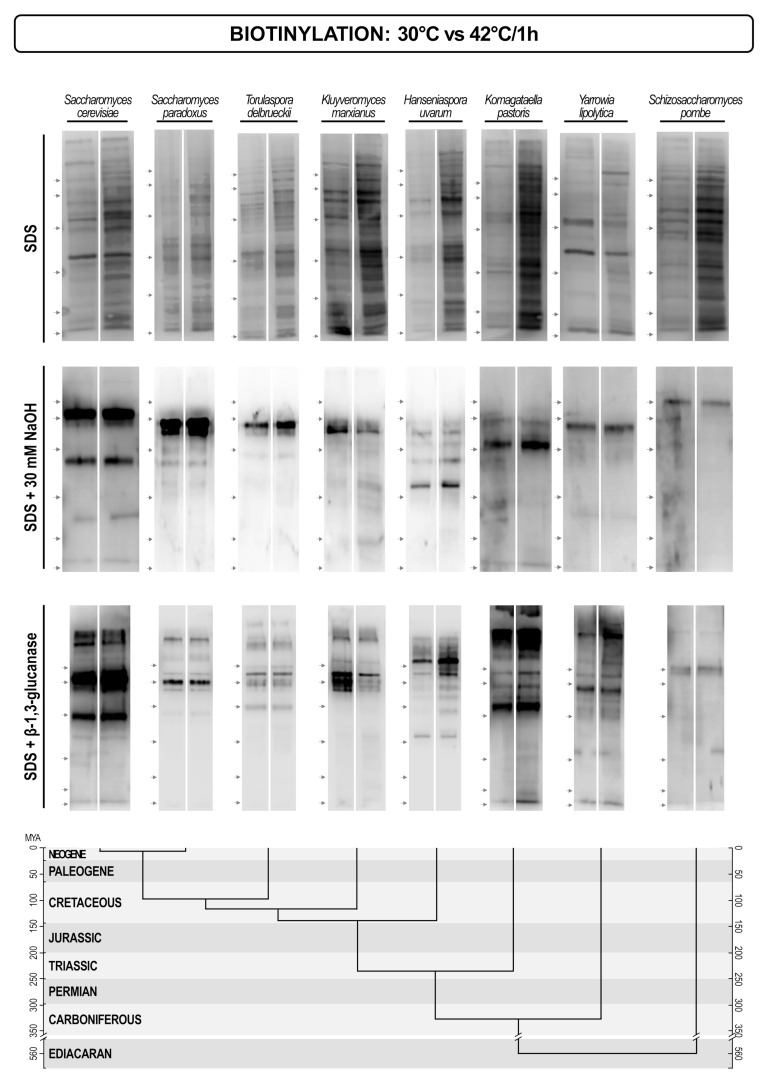
Streptavidin/biotin blots of cell wall proteins of different yeasts extracted by hot SDS (non-covalently attached proteins), alkalis (Pir proteins + Scw4), and glucanases (GPI-anchored proteins). Proteins were extracted from cell walls of yeasts grown at 30 °C (left lane), and then shifted to 42 °C for one hour (right lane). The lower part of the figure presents the evolutionary chronogram of each species. Position of protein molecular mass markers is indicated on the left of each blot with protein sizes identical to those in Figure 6.

Our results indicate that there is a pronounced variability in both protein patterns, and protein concentrations of different yeasts. This difference should result from the intensity of gene expression and their regulation. This, in turn should reflect different environmental conditions and life-styles of different yeasts. To see if a change in the environment would trigger a change in cell wall protein pattern we grew seven yeasts (*S. cerevisiae, S. paradoxus, Torulaspora delbrueckii, Hanseniaspora uvarum, Komagataella pastoris, Yarrowia lipolytica, Schizosaccharomyces pombe*) at 30 °C and then shifted them to 42 °C for one hour. In most yeasts the overall amount of proteins extracted by SDS, but not those extracted by alkalis or glucanase, was higher. This could indicate that there was a general regulatory mechanism that would up-regulate biosynthesis of non-covalently attached proteins, perhaps in relation to their putative functions in cell wall remodeling. Besides, several distinct new proteins appeared in the cell wall of some yeasts, but generally, the protein patterns did not undergo significant changes (Figure 7).

## 4. Discussion

As the outermost structure of the yeast cell, the cell wall is exposed to different environmental factors requiring flexibility and variability that would reflect various habitats and lifestyles of different species. At the same time, cell wall has to fulfill its primary biological role in providing osmotic stability to the cell. Since the latter is mainly insured by the inner glucan layer of the wall it can be expected that the enzymatic apparatus for the biosynthesis and remodeling of β-1,3-, β-1,6-glucan, and chitin are generally conserved among different yeasts. On the other hand, communication with the environment relies mainly on the external cell wall layer composed of mannoproteins. Thus, this component of the wall can be subjected to various evolutionary paths leading to differences in the composition and properties of the external cell surface. It should be noted that mannoproteins contribute to the overall importance of the wall not only through their protein activities but also by forming the external mannan layer that provides the chemical inertness of the cell surface [53] and regulate its porosity [54]. Coronado et al. [40] compared all *S. cerevisiae* genes that are connected with the biosynthesis of the cell wall with genomes of 17 other fungi. Their results indicated that the difference in cell wall related genes correlated well with the taxonomic distance of different fungi, and that the level of conservation of individual genes was strongly dependent on their function. In the first part of this work we performed a similar comparison but at the level of different proteomes of 92 different yeast species/genera. We compared proteins involved in N- and O-glycosylation, GPI synthesis, and the biosynthesis of polysaccharide components of the wall, but primarily focused on the proteins localized within the cell wall that either have roles in wall remodeling, cell-cell communications, or so far unknown functions. Although the comparison methodology was based on protein instead of DNA sequences and the number of species compared was much higher, our results confirmed that the number of orthologues of *S. cerevisiae* cell wall proteins was higher in yeasts that were taxonomically closer while the lowest number of orthologues was found as expected in *Schizosaccharomyces* species (Figure 5). Interestingly, when the conservation of different genes among yeasts was correlated with the way these proteins were incorporated into the cell wall, a pronounced difference could be seen between the non-covalently bound proteins that were rather conserved, and the GPI attached proteins that were quite specific for *S. cerevisiae* with only a few exceptions like the Gas protein family, or Crh proteins. This probably reflects the functions of these proteins. Most non-covalently attached proteins are to some extent structurally related to glycosidases or glycosyl transferases from plants or other microorganisms and, although their exact function is not known it could be assumed that they play roles in the remodeling of wall polysaccharides [55]. Since similar enzymatic reactions are required for these processes in most yeasts their presence in cell walls has to be rather universal. On the other hand, none of these proteins is essential for growth or regular cell cycle indicating that their importance may be related to particular cellular events rather than just growth and cell division. This may explain why *Schizosaccharomyces* species share only a few non-covalently bound proteins with *S. cerevisiae*. In contrast, GPI anchored proteins may have roles that reflect specificities of habitats or lifestyles of different yeasts. Therefore, it seems that rather complex biochemical pathways of GPI and β-1,6-glucan synthesis, proteins’ attachment to GPI, and eventually their translocation to β-1,6-glucan were not designed to attach particular protein(s) or sets of proteins, but rather to enable flexibility in adapting to different environments and conditions. In favor of this could also speak the fact that a rather simple protein modification, addition of the C-terminal GPI-anchoring signal, would result in binding of almost any homologous or heterologous protein in the cell wall through the GPI and β-1,6-glucan linkage [56]. Thus, addition of GPI-anchoring signals to different proteins could be an adaptation strategy in different environments. The exceptions are proteins of the Gas and Crh families known to have the transglycosidase activities required for the proper formation of the cell wall [50], as well as yapsins, aspartic proteases whose role is not yet completely clear. They were reported to participate in proteolytic processing of several cell wall proteins (Scw4, Pir-proteins) and that their activity at least to some extent complements the activity of the Golgi protease Kex2 [14]. At least for Scw4 it has been reported that yapsins also induce its activity [14], but as the exact roles of yapsin substrates are not known, it is difficult to comprehend the role of yapsins, as well. They are, however, very conserved among fungi except in the genus *Schizosaccharomyces*.

The actual composition and activity of cell wall proteins is of course not only the function of the number and similarity of their genes but is also influenced by their transcription rate and regulation. Biotinylation of cell wall proteins, their extraction and electrophoretic analysis enables the comparison of actual protein patterns in the walls. Since transcription can be influenced by growth conditions, stress, or growth phase, we have tried to minimize these influences by growing cells under optimal laboratory conditions and cell wall proteins were extracted from cells at the early logarithmic growth phase. Pronounced differences have been observed among yeasts both in the electrophoretic pattern and in the intensity of different protein bands. Although all fungi share the same components of the GPI-anchoring mechanism, the amount of GPI-anchored proteins was very different. Therefore, not only that this part of the cell wall proteome differed in protein sequences but also the overall quantity of proteins in the walls was different. In contrast, less differences have been observed in the quantity of non-covalently attached (SDS soluble) proteins, although their electrophoretic patterns were different. Clearly, even if the majority of *S. cerevisiae* non-covalently attached proteins had orthologues in most other yeasts in concentrations comparable to those in *S. cerevisiae*, their size was not identical. Pir proteins were present in most budding yeasts (except in *B. adeninivorans* and *D. hansenii*) although usually only one or two homologues were detected compared to as much as four in *S. cerevisiae*. However, in many species Pir-proteins were not visible in streptavidin/biotin blots. The role of these proteins is unknown, but Pir2 of *S. cerevisiae* was reported to be up-regulated under the conditions of stress [57]. However, we did not see an increase of the Pir2 concentration in the wall of *S. cerevisiae*, nor in any other yeast grown at 42 °C (potential increase of the Pir2 concentration in the medium was not tested). Generally, the absence of visible quantities of Pir-proteins in other yeasts may be due to their regulation under different environmental conditions.

The pronounced differences in cell wall protein electrophoretic patterns point out a possibility of application of this method in identification of yeast species since the method is quite simple, it is not time consuming, and it is reproducible. Several results indicate that it can even be used for differentiation among different strains of the same species although this may only be applicable in specific cases. As an example, we observed very different protein patterns in two *Y. lipolytica* strains used. A survey of different *S. cerevisiae* wine yeasts [58] already indicated that cell wall protein patterns may serve as a tool for differentiation among strains. This study showed that flor forming yeasts all share a common pattern of Pir-proteins that differed from the pattern in yeasts unable to form flor [58]. Here we show that different strain specific cell wall protein patterns could be applied for differentiation among strains of other species, as well.

We also found that yeasts that had less GPI-linked mannoproteins in the wall had less mannan compared to glucans. The result was not unexpected but pointed out that the mannoprotein layer in these cells was thinner and that their cell walls may be more permeant. This may be of interest when evaluating different yeasts as tools for biotechnological purposes like the secretion of heterologous proteins or other macromolecules. It also emphasized the possibility for engineering the cell surface by deletions of most abundant GPI-anchored proteins.

Finally, we tried to assess the plasticity of the cell wall protein patterns under the temperature stress conditions. Interestingly, the regulation of only a few proteins seemed to be effected by the temperature shift. Rather, the concentrations of all non-covalently bound cell wall proteins increased. As in the same time the concentrations of covalently bound proteins were not changed, it might indicate that there was a specific regulation of cell wall modifying enzymes at increased temperature. Still, these indications require a more extensive experimental corroboration.

## Data Availability

All the data supporting results published in this manuscript are available from the data repository of the Laboratory of Biochemistry, Faculty of Food Technology and Biotechnology, University of Zagreb.

## Supplementary Materials

The following are available online at https://www.mdpi.com/2309-608X/7/2/128/s1, Table S1: Yeast strains used for the in silico analysis of the cell wall proteomes; Table S2 Yeast strains used in the electrophoretic analysis of the cell wall proteomes.
