# Supplementary material for: Systematic Comparison of Cell Wall-Related Proteins of Different Yeasts
Source: J Fungi (Basel). 2021 Feb 9;7(2):128. doi: 10.3390/jof7020128 (PMC7916363; doi:10.3390/jof7020128)
Supplement: Supplementary file 1 [file jof-07-00128-s001.pdf]

Supplementary Table 1. List of genomes used in the analysis.

Species	Strain	Genbank accession number
Ashbya aceri	FM-2008	GCA_000412225.2
Candida albicans	SC5314	CGD version A22-s07-m01-r125
Candida bracarensis	CBS 10154	GCA_001077315.1
Candida castellii	CBS 4332	GCA_001046935.1
Candida glabrata	ATCC 2001	GCA_010111755.1
Candida nivariensis	CBS 9983	GCA_001046915.1
Debaryomyces hansenii	CBS767	GCA_000006445.2
Eremothecium coryli	CBS 5749	GCA_000710315.1
Eremothecium cymbalariae	DBVPG 7215	GCA_000235365.1
Eremothecium gossypii	ATCC 10895	GCA_000091025.4
Eremothecium sinecaudum	ATCC 58844	GCA_001548555.1
Hanseniaspora osmophila	NRRL Y-1613	GCA_003707715.1
Hanseniaspora uvarum	DSM 2768	GCA_000968475.1
Kazachstania aerobia	NRRL Y-27976	GCA_003708495.1
Kazachstania africana	CBS 2517	GCA_000304475.1
Kazachstania bromeliacearum	NRRL Y-48836	GCA_003708535.2
Kazachstania intestinalis	NRRL Y-48847	GCA_003708845.2
Kazachstania kunashirensis	NRRL Y-27209	GCA_003708465.1
Kazachstania martiniae	NRRL Y-409	GCA_003708925.2
Kazachstania naganishii	CBS 8797	GCA_000348985.1
Kazachstania rosinii	NRRL Y-17919	GCA_003708425.2
Kazachstania saulgeensis	CLIB1764	GCA_900180425.1
Kazachstania servazzii	SRCM 102023	GCA_002214935.1
Kazachstania siamensis	NRRL Y-48842	GCA_003708905.2
Kazachstania solicola	NRRL Y-27207	GCA_003708835.2
Kazachstania spencerorum	NRRL Y-17920	GCA_003708825.2
Kazachstania taianensis	NRRL Y-48846	GCA_003708865.1
Kazachstania telluris	UCD 400	GCA_009394695.1
Kazachstania transvaalensis	NRRL Y-17245	GCA_003708445.2
Kazachstania turicensis	NRRL Y-48834	GCA_003708545.1
Kazachstania unispora	NRRL Y-1556	GCA_003708525.2
Kazachstania viticola	NRRL Y-27206	GCA_003708455.1
Kazachstania yakushimaensis	NRRL Y-48837	GCA_003709265.1
Kluyveromyces aestuarii	NRRL YB-4510	GCA_003707555.1
Kluyveromyces dobzhanskii	NRRL Y-1974	GCA_003705805.2
Kluyveromyces lactis	NRRL Y-1140	GCA_000002515.1
Kluyveromyces marxianus	NBRC 1777	GCA_001417835.1
Kluyveromyces nonfermentans	NRRL Y-27343	GCA_003670155.1
Kluyveromyces wickerhamii	UCD 54-210	GCA_000179415.1
Komagataella pastoris	ATCC 28485	GCA_001708105.1

Lachancea dasiensis	CBS 10888	GCA_900074725.1
Lachancea fermentati	CBS 6772	GCA_900074765.1
Lachancea kluyveri	NRRL Y-12651	GCA_000149225.1
Lachancea lanzarotensis	CBS 12615	GCA_000938715.1
Lachancea meyersii	CBS 8951	GCA_900074715.1
Lachancea mirantina	CBS 11717	GCA_900074745.1
Lachancea nothofagi	CBS 11611	GCA_900074755.1
Lachancea quebecensis	CBS 14088	GCA_002900925.1
Lachancea thermotolerans	CBS 6340	GCA_000142805.1
Lachancea waltii	NCYC 2644	GCA_000167115.1
Metschnikowia bicuspidata var. bicuspidata	NRRL YB-4993	GCA_001664035.1
Metschnikowia pulcherrima	Bath1	GCA_009932455.1
Metschnikowia reukaufii	MR1	GCA_003401635.1
Nakaseomyces bacillisporus	CBS 7720	GCA_001046975.1
Nakaseomyces delphensis	CBS 2170	GCA_001039675.1
Naumovozyma castellii	CBS 4309	GCA_000237345.1
Naumovozyma dairenensis	CBS 421	GCA_000227115.2
Pichia kudriavzevii	CBS5147	GCA_003054405.1
Pichia membranifaciens	NRRL Y-2026	GCA_001661235.1
Saccharomyces cerevisiae	S288C	SGD version: R64-2-1_20150113
Saccharomyces arboricola	H-6	GCA_000292725.1
Saccharomyces eubayanus	CBS 12357	GCA_003327605.1
Saccharomyces jurei	NCYC 3947	GCA_900290405.1
Saccharomyces kudriavzevii	CR 85	GCA_003327635.1
Saccharomyces mikatae	IFO 1815	GCA_000166975.1
Saccharomyces paradoxus	CBS 432	GCA_002079055.1
Saccharomyces sp. 'boulardii'	biocodex	GCA_001298375.2
Saccharomyces uvarum	U3	GCA_002242645.1
Schizosaccharomyces japonicus	yFS275	GCA_000149845.2
Schizosaccharomyces pombe	972h-	GCA_000002945.2
Tetrapisispora blattae	CBS 6284	GCA_000315915.1
Tetrapisispora fleetii	NRRL Y-27350	GCA_003707605.1
Tetrapisispora iriomotensis	NRRL Y-27309	GCA_003705975.1
Tetrapisispora namnaoensis	NRRL Y-27982	GCA_003705985.1
Tetrapisispora phaffii	CBS 4417	GCA_000236905.1
Torulaspora delbrueckii	COFT1	GCA_003013175.1
Torulaspora franciscae	NRRL Y-17532	GCA_003705175.2
Torulaspora maleeae	CBS 10694	GCA_003708055.2
Torulaspora microellipsoides	NRRL Y-1549	GCA_003707085.1
Torulaspora pretoriensis	CBS 2187	GCA_012851205.1
Vanderwaltozyma polyspora	DSM 70294	GCA_000150035.1
Wickerhamomyces anomalus	NRRL Y-366-8	GCA_001661255.1

Yarrowia lipolytica	DSM 3286	GCA_014490615.1
Yueomyces sinensis	NRRL Y-17406	GCA_003707995.1
Zygosaccharomyces bailii	IST 302	GCA_900162805.1
Zygosaccharomyces bisporus	NRRL Y-12626	GCA_003707595.1
Zygosaccharomyces kombuchaensis	NRRL YB-4811	GCA_003705955.1
Zygosaccharomyces mellis	Ca-7	GCA_005406105.1
Zygosaccharomyces rouxii	CBS 732	GCA_000026365.1
Zygotorulaspora florentina	NRRL Y-1560	GCA_003671575.2
Zygotorulaspora mrakii	NRRL Y-12654	GCA_003671565.1

Supplementary Table 2. List of strains used in labelling and detection of cell wall proteins

Species	Strain designation		Source
	ACBR	CBS	
Blastobotrys adeninivorans	HA1092^{T}	CBS8244^{T}	Soil, Netherlands, Wageningen
Debaryomyces hansenii	HA574	-	Excrement of birds in a zoo
D. vindobonensis	HA1076^{T}	CBS11666^{T}	Wastewater treatment plant, Austria, Vienna
Hanseniaspora osmophila	HA1225	CBS314^{T}	Muscatel grape, Russia, Crimea
H. uvarum	HA1216^{T}		
Kluyveromyces lactis	HA118	CBS2359	creamery, USA
K. marxianus	HA731^{T}	CBS834^{T}	kefyr grain, Netherlands
Metschnikowia biscupidata	HA672^{NT}	CBS5575^{NT}	sporocysts of *Diplostomum flexicaudum* (trematode), in digestive gland of snail
M. pulcherrima	HA665^{T}	CBS5833^{T}	Berries of *Vitis labrusca*; USA, California
M. reukaufii	HA666^{T}	CBS 5834^{T}	Flower of *Epilobium angustifolium*, Canada
Pichia kudriavzevii	HA892^{T}	CBS5147^{T}	fruit juice
P. membranifaciens	HA895^{T}	CBS107^{T}	unknown
S. cerevisiae	HA2779	-	BY4741, derivative of S288C strain
S. cerevisiae v. boulardii	HA282	CBS5926	Fruit, Indochina
S. paradoxus	HA390	CBS406	Exudate of Quercus sp., Netherlands
Schizosaccharomyces japonicus	HA116^{T}	CBS103^{T}	concentrated grape juice, USA
Sch.pombe	HA983^{T}	CBS357^{T}	cane-sugar molasses, for rum making, Jamaica
Torulaspora delbrueckii	HA688^{NT}	CBS1146^{NT}	unknown
Yarrowia lipolytica	HA990^{T}	CBS6124^{T}	maize processing plant; USA, Illinois
Y. lipolytica	HA826	-	surface ripened soft cheese, Austria

T, type strain; NT, neotype strain; ACBR, Austrian Center of Biological Resources and Applied Mycology, Muthgasse 11, 1190 Vienna, Austria; CBS, CBS-KNAW Fungal Biodiversity Centre, Uppsalalaan 8, 3584 CT Utrecht, The Netherlands
